# Female-Released Sex Pheromones Mediating Courtship Behavior in *Lysiphlebus testaceipes* Males

**DOI:** 10.1673/031.013.5301

**Published:** 2013-06-14

**Authors:** Mirella Lo Pinto, Benedetta Cangelosi, Stefano Colazza

**Affiliations:** Department of Agricultural and Forest Sciences (SAF), University of Palermo, Viale delle Scienze, 13, 90128 Palermo, Italy

**Keywords:** Braconidae, close-range communication, cuticular compounds, mating, nonpolar fraction, polar fraction, wing fanning

## Abstract

Ethological aspects and chemical communication at close-range between the sexes of *Lysiphlebus testaceipes* Cresson (Hymenoptera: Braconidae) have been investigated through behavioral bioassays and chemical analysis. The attractiveness toward males of whole-body extracts of females and males in hexane and acetone was evaluated, adopting male fanning behavior as a key behavioral component. Also, the activity of polar and nonpolar fraction of female-body extract in hexane obtained using solid-phase extraction technique was investigated. In order to identify cuticular compounds, male and female whole-body extracts with hexane and acetone were analyzed by gas chromatography-mass spectrometry. The results showed that males exhibit a behavior including 4 phases when exposed to virgin females: premount, mount, copulation, and post-copulation. A preliminary courtship of the male included wing fanning, an extension and vibration of the wings for 1 to 2 seconds. Also, some original aspects not described for other species were carried out. The average duration of the entire sequence of events was 138.80 ± 19.51 sec. Also, males displayed significantly more wing fanning behavior in response to female whole-body hexane extracts (70.83%) than female whole-body acetone extracts (33.3%). Furthermore, males did not respond to male-body extracts or to the control (pure hexane and acetone), suggesting that the sex pheromone is composed of cuticular hydrocarbons that are also involved in the male courtship behavior. When hexane extracts of whole females were fractionated on silica gel and exposed to males, more activity was recorded for the nonpolar fraction (50.0%) than the polar fraction (27.7%), but no significant statistical difference was found. Significant differences were detected comparing the control (not fractionated extract) with the polar fraction, but not with the nonpolar fraction. A homologous series of *n*-alkanes with chain lengths from C19 to C30 carbon atoms was identified and quantified in the solvent extracts of wasp males and females. Between male and female extracts, there was a statistically significant difference in the average quantity of some of these hydrocarbons, such as C_27_, C_28_, and C_29_.

## Introduction

*Lysiphlebus testaceipes* Cresson (Hymenoptera: Braconidae) is a generalist parasitoid, having an extremely broad range of hosts (Mackauer and Starý 1967) and different preferences for different aphid hosts on various plants (Jackson et al. 1970). This species originated in North and Central America (Mackauer and Starý 1967), and was originally introduced from Cuba to Southern France in 1973 for the biological control of *Toxoptera aurantii* and *Aphis spiraecola* (*= citricola)* (Starý et al. 1988a, 1988b). It gradually becomes widespread both in the west and east coastal areas of the Mediterranean basin (Starý et al. 2004). *L. testaceipes* is known to contribute to aphid control by causing both a direct mortality of the host and a decrease in its reproductive rate (Spencer 1926; Giles et al. 2003).

Previous in-field research showed evidence for a long-distance sex pheromone of *L. testaceipes* virgin females that attracted conspecific males in the first 3 days of female life, with greater intensity in the early hours on the first day and in the afternoon on the third day (Lo Genco et al. 2005). The influence of the female mating status on the male attraction and foraging behavior was also highlighted; in laboratory, males were significantly more attracted to virgin females than to mated females when bioassayed in a *Y*-tube olfactometer (Lo Pinto et al. 2002). In field, virgin females stayed motionless more often and for longer periods than mated females, and attacked aphids at a lower rate (Fauvergue et al. 2008).

Sexual behavior has been described in many parasitoid species (Evans and Matthews 1976; van den Assem 1976; Gordh and DeBach 1978; Lo Pinto 1989; Rüther et al. 2000; Pandey and Singh 2004; González and Matthews 2005), and the presence of sex pheromones have been reported in some Braconidae, such as *Opius alloeus* (Boush and Baerwald 1967), *Apanteles medicaginis glomeratus* (Obara and Kitano 1974), *Apanteles medicaginis melanoscelus* (Weseloh 1981), *Cotesia rubecula* (Field and Keller 1994), *Cotesia flavipes* (Kimani and Overholt 1995), *Praon volucre* (Nazzi et al. 1996), *Fopius arisanus* (Quimio and Walter 2000), and *Glyptalantes flavicoxis* (Danci et al. 2006), as well as in other families (Pompanon et al. 1997; Cormier et al. 1998). Although sex pheromones have been widely reported for many insects, they have been identified only in a few species, such as *Itoplectis conquisitor* (Robacker and Hendry 1977), *Syndipnus rubiginosus* (Eller et al. 1984), *Ascogaster reticulatus* (Kainoh et al. 1991), *Cardiochiles nigriceps* (Syvertsen et al. 1995), *Ascogaster quadridentata* (DeLuryet al. 1999), *Roptrocerus xylophagorum* (Sullivan 2002), and *Nasonia vitripennis* (Steiner et al. 2007). Some works showed that short-range sexual communication could be regulated by cuticular compounds (Ferveur 2005; Howard and Blomquist 2005). The cuticle of insects is characterized by a long-chain of hydrocarbons, acids, alcohols, esters, aldehydes, and ketones, which protects them from dehydration (Gibbs 1998) and may play a role in inter- and intra-specific communication (Stoffolano et al. 1997; Steinmetz et al. 2003; Howard and Blomquist 2005; Ruther et al. 2011; Salerno et al. 2012).

In *L. testaceipes*, though the attractiveness of virgin females to males at long and short distances has been found, the sex pheromone involved in the male response has not yet been identified and characterized chemically. Therefore, this research was conducted to elucidate the female-released close-range sex pheromone and to identify the chemical compounds present in the body's cuticle that stimulate the male's courtship behavior. In particular, the mating behavior of *L. testaceipes* was studied through the definition of the characteristic stages and steps of the male courtship. Also, the role played by the cuticle hydrocarbons as sex pheromones was investigated by evaluating the attractiveness to males of hexane and acetone body extracts of both sexes. Furthermore, the attraction activity of polar and nonpolar fraction of female-body extract in hexane to males has been detected.

## Materials and Methods

### Insects

Parasitoids were mass-reared on *Aphis gossypii* (Hemiptera: Aphididae) on cu-cumber plants, *Cucumis sativa* (Cucurbitales: Cucurbitaceae). Plants were kept in a box in a greenhouse not conditioned with natural light. When plants had grown 15 to 20 cm high with 3 to 4 real leaves, they were moved to another box and infested with *A. gossypii*. The rearing of parasitoids was carried out in a third box in the same greenhouse used for the host. In order to obtain standardized young adults, parasitized aphids were isolated at the mummy stage in corked glass vials (8 × 60 mm) and kept in an environmental room at 26° C, 70 ± 5% RH, and under a 16:8 L:D photoperiod. Emerging adults were fed with a honey-water solution and used in experiments. Some of these adults were put in single pairs, male and female, in glass vials until copulation, and were introduced in the box with infested plants to maintain the parasitoid rearing.

### Mating behavior

Twenty pairs of unmated one- to two-dayold adults were held singly in gelatin capsules (diameter = 0.7 cm; length = 2 cm) and their behavior was recorded with a video camera (Hitachi KP-D40, www.hitachi.com) connected to a monitor (Panasonic TC-1470Y, www.panasonic.com) and a VCR (Sharp VC-GH600DSM, sharp-world.com). To acclimatize insects to the test's abiotic conditions, each parasitoid wasp was kept isolated in corked glass vials (8 × 60 mm) for 5 minutes in the laboratory room before the test. Then, individuals of opposite sex were introduced in the gelatine capsules, 1 pair per capsule, and used in the test. The pairs were observed up to 2 minutes following the copula for a maximum of 10 minutes. Individuals that did not mate were excluded from analyses. Recordings took place between 09:00 and 14:00 under laboratory conditions (25 ± 1° C, 70 ± 10% RH) and were transferred through a board of image acquisition from the videotape to a PC equipped with a video editing software (Pinnacle, www.pinnaclesys.com) for data processing in relation to the time (mean ± SE) spent in the performance of the events of each mating behavior phase (pre- and post-mount, mount, copulation, etc.) and definition of characteristic behavioral aspects.

### Chemical analysis of *L. testaceipes* cuticular linear hydrocarbons

Unmated, 1- to 2-day-old females (20) and males (20) were killed by freezing at -20° C for about 1 hr, and placed into vials (4 mL) containing hexane or acetone (200 µl) for 1 hour. Extracts were kept in freezer at 20° C until they were used for the experiments (within 36 hours).

To identify and quantify the *n*-alkanes of the cuticle, whole-body hexane extracts of males and females of *L. testaceipes* were analyzed by a Hewlett-Packard 5890 (www.hp.com) gas chromatography-mass spectrometry system interfaced with an HP 5973 quadrupole mass spectrometer detector and a HP5-MS column (5% diphenyl95% dimethylpolysiloxane; 30 m × 0.2 mm ID, 0.25 µm film thickness; J&W Scientific, Folsom CA, USA). Injections of hexane extracts took place in a chamber for injecting, which had an inner diameter of 4 mm and was packed with glass wool. The tests were carried out in splitless mode.

The carrier gas was helium (99.999% purity), with a constant flow of 1 mL per min-^1^. The chamber injector temperature was set at 270° C, and an oven temperature program was set at the following: initial temperature 150° C and isothermal for 2 minutes, then increased 5° C/min to 280° C and isothermal for 10 minutes. The ionization energy for molecular fragmentation was 70 eV, recording the mass spectra from 42 to 550 AMU. The hydrocarbons were identified by a qualitative comparison of experimental mass spectra with NIST 98 database (http://www.nist.gov/srd/nist1.htm). A mixture of *n*-alkanes (nC19-nC36) (all 99% purity; Fluka, Sigma-Aldrich, www.sigmaaldrich.com) in hexane served as the external standard to identify and quantify each compound.

### Petri dish Bioassays

**Whole body solvent extracts of males and females.** Bioassays to evaluate the attractiveness of body extracts were conducted using unmated males in the absence of visual and tactile stimuli, and considered response behavioral characteristics of males, such as the wing-fanning behavior. Six treatments were tested: malebody extracts in hexane and in acetone, female-body extracts in hexane and in acetone, pure hexane, and pure acetone. For each treatment, 10 µl of extracts were put on filter paper discs in Petri glass dishes (ø = 5 cm) using a Pasteur pipette. Additional Petri glass dishes served as a control stimulus, with solvent (hexane or acetone) applied in the same way as the treatment. After the solvent had evaporated (7 min), a virgin, 1- to 2-day-old male was released into the Petri dish, which was sealed with a lid, and the male was observed for 1 min. Wing fanning behavior was recorded. For each filter paper disc, the responses of 3 males were recorded. The order of the presentation of the 6 types of treatments was randomized within each day of the experiment. In total, 24 replicates for each treatment were done. Experiments were conducted between 9:00 and 14:00 at 25 ± 1°C and 60 to 70% RH.

**Table 1.**
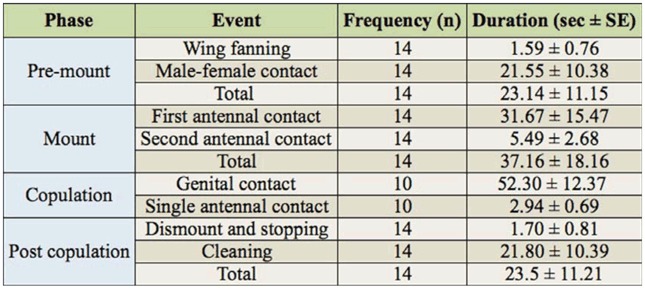
Frequency (n) and duration (average ± standard error) of the behavioral events of the 4 phases observed (14 pairs) in the courtship sequence of *Lysiphlebus testaceipes*.

**Fractions of body hexane extracts of females.** Fractions of female extracts were made using only the whole-body hexane extracts because the whole-body extracts elicited the majority of males performing the wing fanning. Hexane extracts of *L. testaceipes* females were fractionated by liquid chromatography, and the resulting polar and nonpolar fractions were bioassayed. The 2 fractions were produced using solid-phase extraction technique. A column packed with silica gel (LC-SI Supel clean SPE tubes, 1 mL; Supelco, Sigma-Aldrich) was rinsed by 5 mL methanol and then 5 mL hexane solution with 5% methanol. Hexane extracts (500 µl) of previously obtained females were collected in a volumetric flask, and hexane was added to the extracts to make the volume 5 mL. 2.5 mL of this sample was made to flow through the column, while the remaining aliquot was stored in a freezer (-20° C) for control in subsequent bioassays. The liquid that flowed into the column was collected, and hexane was added until a volume 5 mL was reached. Thus, nonpolar fraction was obtained for the bioassay. To obtain the polar fraction, the column was again rinsed, eluting 5 mL hexane with 5% methanol, and then 5 mL acetone was made to flow through the column. The liquid was collected, and acetone was added until the volume reached 5 mL. In bioassays, 3 treatments were tested: polar fraction, nonpolar fraction, and not fractionated (control) female-body extracts. Bioassays to evaluate the attractiveness to males of the fractions of extracts were performed using the same procedure previously described. Each treatment was replicated 18 times.

### Statistical analyses

Data obtained from bioassays were analyzed using Pearson's *χ*^2^ test considering the real values reported. In relation to the identification of cuticular hydrocarbons, the comparison of the quantity of hydrocarbons present in the cuticle of males and females was performed using the StudentNewman-Keuls test for all-pair wise (SigmaStat Statistical Software 1997). The quantity of hydrocarbons was expressed in ng mg-1 analyte per weight of the sample.

## Results

### Mating behavior

Recordings on pairs showed that the courtship and mating behavior of *L. testaceipes* occured in stereotyped, ethological sequences consisting of the phases premount, mount, copulation, and post-copulation, each characterized by peculiar aspects (Table 1). In the pre-mount phase, a male and female walked in the arena, exploring the substrate with their antennae. When the male perceived the female, he performed a preliminary courtship called wing-fanning, which consisted of either extending and vibrating his wings for 1 to 2 seconds as he was walking (85.7%), or standing with his antennae raised and spread with his wings raised and fluttered (14.3%). Subsequently, the male touched with his antennae the head, mesosoma, or gaster of the female, which was still moving. In 42.8% of cases, the male alternated between wing-fanning and antennal contact. The female showed her receptivity to courtship of the male by stopping and remaining motionless with her antennae spread and pointed upwards, with the tips slightly backwards. This phase lasted on average 23.14 ± 11.15 sec (n = 14).

During the mount phase, once the female was motionless, the male holding his wings open mounted the female, positioning his fore and middle tarsi on the mesosoma, and the hind tarsi on the gaster of the female. Then, he placed his mouthparts in contact with the female's head and mesosoma, establishing characteristic antennal contact. The male then rapidly drummed its antennae, alternately stroking his left and right antenna on the corresponding (left and right) female antenna. Just before the next phase of copulation, a second type of antennal contact was shown, as the male performed the antennal drumming more slowly, with longer intervals between alternating strokes. Simultaneously, he slid backwards, curling his gaster under that of the female while holding his fore, middle, and hind legs on her mesosoma, gaster, and substrate, respectively. During this phase, which lasted 37.16 ± 18.16 sec (n = 14), the female remained in the same position. However, in 28.6% of cases, the female was un-receptive and held her antennae downwards, keeping her gaster lowered and not permitting the copulation phase.

In the copulation phase (average duration 52.30 ± 12.37 sec, n= 10), the male established genital contact with the female and leaned the upper part of his body backward, with only his forelegs on the female gaster and his wings raised. During this phase, a third type of antennal contact was observed; the male continued the performance of antennal drumming observed in the last part of the mount phase, but stopped the antennal movements intermittently 3 to 5 times for 2 to 3 seconds.

**Table 2.**
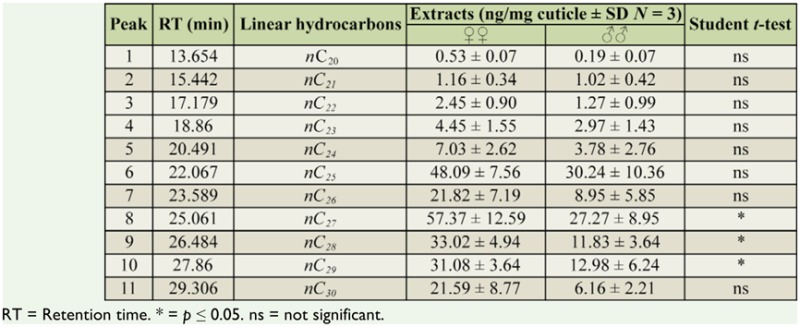
Cuticular hydrocarbons identified in whole-body hexane extracts of *Lysiplebus testaceipes*. Means and standard errors calculated for 3 cuticular extracts.

After copulation, the post-copulation phase (average duration 23.5 ± 11.21 sec, n= 14) was characterized by the male's dismount and cleaning by both sexes. The male dismounted from the rear, stopped at short distance from the female, and started to clean his antennae and head with his forelegs, and his body with his mouthparts. The female performed similar cleaning, but sometimes this behavior was not observed, as the female sometimes began to walk away at the end of the copula. At the end of the cleaning, both sexes walked away from each other. Further courtship or attempts to mate by the male were not observed, though contact occurred between individuals during their movements. The entire sequence of events lasted on average 138.80 ± 19.51 sec(n=10).

**Figure 1.**
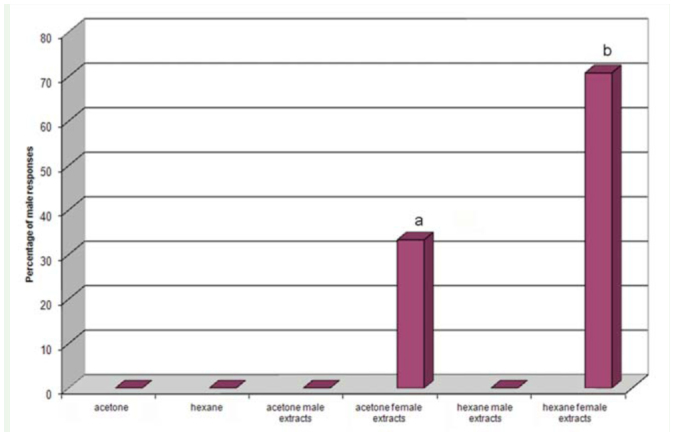
Percentage of male *Lysiphlebus testaceipes* responses to acetone, hexane, and acetone and hexane extracts of both male and females. High quality figures are available online.

**Figure 2.**
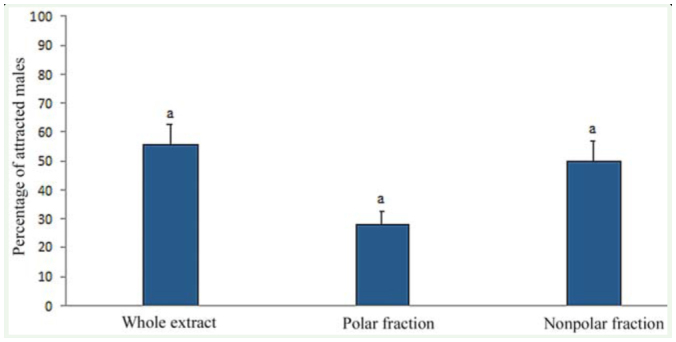
Percentage of male *Lysiphlebus testaceipes* attracted to whole extract, polar fraction, and nonpolar fraction. High quality figures are available online.

### Chemical analysis of *L. testaceipes* cuticular linear hydrocarbons

Chemical analysis to identify and quantify cuticular hydrocarbons using whole-body hexane extracts of males and females of *L. testaceipes* showed a homologous series of *n*-alkanes with chain lengths from C19 to C30 carbon atoms (Table 2). Between male and female extracts, there was a statistically significant difference (*p* < 0.05) in the average quantity of some of these hydrocarbons, such as C27 (*p* < 0.05), C28 (*p* < 0.05), and C_29_ (*p* < 0.05).

### Petri dish Bioassays

**Whole body solvent extracts of males and females.** In bioassays to evaluate attractiveness of body extracts, males displayed significantly more wing-fanning to female whole-body extracts in hexane than to those in acetone (Figure 1), while they did not respond to either the malebody extracts or the control (pure hexane and acetone). In particular, hexane and acetone extracts of females induced response in 70.83% and 33.30% of males tested, respectively. Differences between the 2 responses were significant χ2 = 6.76; *p* ≤0.05; df = 1).

**Fractions of body hexane extracts of females.** In bioassays to compare the activity of the polar and nonpolar fraction of the female-body extracts in hexane, males exhibited wing-fanning when exposed to both fractions and the control (not fractionated extract) (Figure 2). In particular, responses to polar and nonpolar fractions and the not fractionated extract were elicited in 27.7%, 50.0%, and 55.5% of males tested, respectively. Although major activity was detected as a result of the nonpolar fraction, no statistically significant difference was found between the responses to the 3treatments (polar fraction, nonpolar fraction and control) (χ2 = 3.15; *p* = 0.21; df =2).

## Discussion

This study revealed the existence of a short-range sex pheromone produced by virgin females of *L. testaceipes* that elicits male courtship in the absence of visual/tactile cues normally associated with a female. Chemical stimuli are known to play an important role in short-distance communication between males and females (Hardy et al. 2007), and are important in inducing female receptivity (van den Assem et al. 1980). In *L. testaceipes*, the courtship and mating behavior occur according to welldefined sequences similar to those observed in most braconid species (Hardy et al. 2007), which include different components eliciting progressive behavioral steps. Acts such as wing fanning, antennal drumming, or mouthpart extrusions serve to induce female receptivity (Ruther et al. 2010). The switch from latent to overt receptivity is generally indicated by the female raising her gaster and opening her genital orifice (Hardy et al. 2007). In our observations, the performance of the male in the pre-copulatory phase induced receptivity in the female, which assumed a typical posture. Nevertheless, the 3 different types of antennation observed during the mount and copulation phases are not described for other species. These antennal contacts exhibited by males are probably related to the issue of chemical signals to stimulate the receptivity of the female. Moreover, the male always performed the wing-fanning before mounting, unlike what was reported in a previous study on this species (Marullo 1987). This behavior can be a visual stimulus for the female, indue-ing receptivity (Kitano 1975; Gordh and DeBach 1978; van den Assem 1986) and increasing the likelihood of attracting a mate (Benelli et al. 2012), or can serve to improve the orientation toward the odorous source (Vinson 1972, 1978; Danci et al. 2010).

Our results support the hypothesis that the short-range sex pheromone eliciting the courtship of the male is composed of cuticular hydrocarbons of the female's body. The pheromone stimulation of male courtship behavior in the absence of further stimuli has been reported in other Hymenoptera species (Obara and Kitano 1974; Kitano 1975; Robacker et al. 1976; Vinson 1978; Yoshida 1978; Takahashi and Sugai 1982; Shu and Jones 1993). These odors induce male wing-fanning. All cuticular hydrocarbons were long-chain alkanes removed from whole insects by steeping them in hexane, and had been previously identified in the cuticular hydrocarbons of other insects (Blomquist et al. 1987; Carlson et al. 1998). The significant difference between the responses of males to female hexane extracts and to female acetone extracts suggests that short-range communication of *L. testaceipes* cuticular hydrocarbons may play an important role, as they do in several orders of insects (Howard 1993). In our study, *L. testaceipes* males responded to whole-body extracts of females, but did not respond to either extracts of males or the control (pure solvent). This behavioral response of males was supported by chemical analysis, from which emerges a significant difference between the average amount of linear hydrocarbons detected in males and females. Similar results were reported for *Spalangia endius* (Nichols et al. 2010) and *R. xylophagorum* (Espelie et al. 1996),suggesting that qualitative and quantitative differences in the cuticular hydrocarbon composition of males and females might play a role in close-range mate recognition.

In this study, the activity of sex pheromone was obtained using the entire body of females, as a single body region is not identified as the site of pheromone production. Several works report that sex pheromones in hymenopteran species originate in the abdomen (Tagawa 1977; Vinson 1978; Yoshida 1978; Askari and Alishah 1979; Ruther et al. 2000), the head and/or thorax or mesosoma (Takahashi and Sugai 1982; Kainoh and Oishi 1993; Salerno et al. 2012), or both regions (Vinson 1972), and pheromone-producing glands have been identified in both the abdomen and head (Tagawa 1977; Wesel oh 1980; Syvertsen et al. 1995; Jones 1996).

Although males showed a greater tendency to respond to nonpolar fraction than polar fraction, no statistical difference was found between the number of responses obtained from the 2 fractions and the control (not fractionated extracts). This result suggests that active compounds could be included in both fractions, and there is a synergistic action of both polar and nonpolar compounds, as reported in the literature for another species of parasitoids, *Eriborus terebrans* (Shu and Jones 1993), for which active compounds in the sexual attraction included in both fractions were identified. A recent study on *Trissolcus brochymenae* reported that polar components of the sex pheromone play a major role in influencing male behavior (Salerno et al. 2012).

Future studies on the identification of the short-range *L. testaceipes* sex pheromone components of both polar and nonpolar fractions, and studies on which chemicals are of particular importance on the bioactive hydrocarbons profile from a single body region, are necessary.
